# EEG Slowing and Axial Motor Impairment Are Independent Predictors of Cognitive Worsening in a Three-Year Cohort of Patients With Parkinson's Disease

**DOI:** 10.3389/fnagi.2020.00171

**Published:** 2020-06-18

**Authors:** Vitalii V. Kozak, Menorca Chaturvedi, Ute Gschwandtner, Florian Hatz, Antonia Meyer, Volker Roth, Peter Fuhr

**Affiliations:** ^1^Neurology and Neurophysiology, University Hospital of Basel, Basel, Switzerland; ^2^Mathematics and Computer Science, University of Basel, Basel, Switzerland

**Keywords:** EEG, Parkinson's disease, cognitive impairment, prediction, axial motor impairment

## Abstract

**Objective:** We aimed to determine whether the combination of two parameters: (a) score of axial impairment and limb rigidity (SAILR) with (b) EEG global relative median power in the frequency range theta 4–8 Hz (GRMPT) predicted cognitive outcome in patients with Parkinson's disease (PD) better than each of these measures alone.

**Methods:** 47 non-demented patients with PD were examined and re-examined after 3 years. At both time-points, the patients underwent a comprehensive neuropsychological and neurological assessment and EEG in eyes-closed resting-state condition. The results of cognitive tests were normalized and individually summarized to obtain a “global cognitive score” (GCS). Change of GCS was used to represent cognitive changes over time. GRMPT and SAILR was used for further analysis. Linear regression models were calculated.

**Results:** GRMPT and SAILR independently predicted cognitive change. Combination of GRMPT and SAILR improved the significance of the regression model as compared to using each of these measures alone. GRMPT and SAILR only slightly correlate between each other.

**Conclusion:** The combination of axial signs and rigidity with quantitative EEG improves early identification of patients with PD prone to severe cognitive decline. GRMPT and SAILR seem to reflect different disease mechanisms.

**Significance** Combination of EEG and axial motor impairment assessment may be a valuable marker in the cognitive prognosis of PD.

## Introduction

Since dementia in Parkinson's Disease (PD) dramatically worsens the course of the disease (Levy et al., 2002; Aarsland and Kurz, 2010; Aarsland et al., 2017) early and correct identification of the patients at high risk of developing dementia over the long term course of the disease is highly relevant. Finding reliable prognostic markers of cognitive decline in PD is therefore a challenging and crucial task, which has led to many suggestions of possible important factors (Mollenhauer et al., 2014).

Some researchers suggested that a combination of different prognostic markers—sometimes this combination is referred to as ≪composite marker≫—allows to identify patients with PD who have a risk of dementia, better than a single marker (Sonnen et al., 2008; Shi et al., 2010; Liu et al., 2017). To explain this finding the researchers made the assumption that the analysis of various pathological aspects of PD (e.g., genetic susceptibility, protein overproduction and accumulation, cortical function etc.) increases the precision of calculation of the dementia risk. For instance, evaluation of body temperature, chest X-Ray, and blood parameters will surely better predict the course of pneumonia than the analysis of body temperature alone.

Another important issue in the identification and selection of component markers is avoiding multicollinearity. Multicollinearity in statistical calculation, i.e., regression models, occurs when some factors are correlated not just to the dependent variable, but also to each other. Thus, such redundant factors significantly impair the precision of the statistical calculation (for details, see Allen, 1997).

Therefore, each of the component markers should reflect non-redundant information on the disease otherwise the reason to include such markers in the risk calculation is questionable.

There is solid evidence that the slowing of EEG, which refers to reduced EEG spectral power in ranges above 8 Hz (such as alpha, beta), and increased spectral power below 8 Hz (theta, delta), is a risk marker of cognitive decline in PD (Caviness et al., 2015; Aarsland et al., 2017; Babiloni et al., 2018). Additionally, Gago et al. (2009) reported that worsening of axial motor signs is a risk factor of dementia in PD. In a meta-analysis by Xu et al. (2016) higher values of part III of the Unified Parkinson's Disease Rating Scale (UPDRS-III) were associated with the dementia risk in patients with PD.

In our study, we aimed to (1) replicate the evidence on quantitative EEG (qEEG) and axial motor signs as risk factors for PD related cognitive decline; (2) check if qEEG and axial motor signs are independent risk factors of PD related cognitive decline.

Our hypothesis was that a combination of qEEG and axial motor signs is a better prognostic marker of cognitive decline, than each of these two factors alone.

## Materials and Methods

### Patients

We retrospectively selected participants from the research database of the Department of Neurology and Neurophysiology of the University Hospital Basel (please see Section Selection Criteria for selection criteria). This database has prospective records of patients with PD, who are investigated for risk factors of cognitive decline. Patients are examined on inclusion and then re-examined after definite time periods. Examinations consist of clinical evaluation, cognitive testing, and EEG recording, were obtained within 2 weeks.

All patients were fully informed about the character of the study and provided their informed written consent on collection and analysis of their data. The study protocol was approved by the local ethics committee (Ethikkommission beider Basel ref. no.: 135/11).

### Selection Criteria

We selected patients according to the following criteria:

a) PD diagnosis according to Unified PD Brain Bank criteria Hughes et al. (1992).
b) no history of stroke, multiple sclerosis, Alzheimer's disease or other severe disorders of the nervous system;
c) examinations performed on inclusion and after 3 years (duration of observation 3 years);
d) no dementia at initial examination.

### Clinical Evaluation

We assessed the patients with part III of the Unified Parkinson's Disease Rating Scale (UPDRS-III). Levodopa equivalents of daily dose (LEDD) was calculated according to Tomlinson et al. (2010).

### Score of Axial Impairment and Limb Rigidity (Axial Motor Signs)

In addition to using the UPDRS-III score as a predictor, the score of axial motor signs was quantified based on prior reports (Bejjani et al., 2000; Gago et al., 2009) as the sum of items speech, rigidity (neck and all limbs), postural stability, posture, and gait.

### Cognitive Testing and Composite Cognitive Score

Patients underwent comprehensive neurocognitive assessment with a battery of 14 psychological test variables, performed by experienced neuropsychologist. Cognitive evaluation was performed in individual sessions. Tests are described in Strauss et al. (2006) and Tewes (1991) if not indicated otherwise. The cognitive test variables were transformed to a z-score based on age, sex, and education normative database (Berres et al., 2000). An overall test score “global cognitive score” was calculated by averaging z-scores of all test variables regardless of the domain (see Table 1).

**Table 1:** Neurocognitive Test Battery, **TAP**, Test battery of Attentional Performance; **WCST**, Wisconsin Card Sorting Test.

**I Attention** • Stroop (color words, Stroop, 1935) • Trail Making Test A (Reitan, 1958)	**II Executive Functions** • WCST (number of errors, Nelson, 1976) • Trail Making Test B divided by A (Reitan, 1958) • Stroop (Time interference, Stroop, 1935)
**III Verbal Fluency** • Semantic Fluency (Isaacs and Kennie, 1973) • Phonemic Fluency (Thurstone, 1948)	**IV Visuospatial Functions** • Block Design Test (Härtig et al., 2000) • Rey Complex Figure Copy (Rey, 1941)
**V Memory** • California Verbal Learning Test (Savings, Discriminability, Delis et al., 1987)	**VI Working memory** • Corsi blocks (Härtig et al., 2000) • TAP, divided attention (omissions, Zimmermann and Fimm, 2007)

### EEG

A continuous eyes-closed resting state 256-channel-EEG of ~15 min was recorded in each patient (Netstation 300; EGI Inc. Oregon, USA). Sampling rate was 1000 Hz and reference electrode was Cz, referenced to average. Artifact free segments of at least 35 s were visually selected. EEGs were filtered (2,500 order least square filter; band pass: 0.5–70 Hz, notch: 50 Hz) and bad electrodes were automatically detected [TAPEEG; (Hatz et al., 2015)]. The eyes closed segments were of 12 min duration.

Artifacts such as ECG and eye blinks were detected and removed by application of an independent component analysis (ICA). Bad channel detection as well as ICA was carried out using the FASTER and FieldTrip algorithms integrated in the TAPEEG software. Channels with bad activations were automatically detected and interpolated by spherical spline method.

Frequency analysis was performed [“Welch”-method (Welch, 1967), sliding window of 4 s with 80% Hanning window and detection of bad windows with automated routines (Hatz et al., 2015)].

Relative power was obtained for five frequency bands: delta (1–4 Hz), theta (4–8 Hz), alpha1 (8–10 Hz), alpha2 (10–13 Hz), and beta (13–30 Hz), by calculating the ratio of the signal power within a frequency band to the total signal power (1–30 Hz).

Global relative median power in the theta band is the variable of interest, where the median power in the theta band is measured relative to the power in the other bands in a specific patient at baseline. The global value is an average over the powers obtained over all electrodes and regions and is logit transformed.

### Statistics

Continuous variables were visually inspected and statistically tested by Kolmogorov–Smirnov test for normality of the distribution.

In a first step, to check for the multicollinearity, respectively, for the independency of the variables, we analyzed correlations between all predictors. Second, we used general linear regressions with Akaike Information Criterion (AIC) based backward stepwise elimination to analyze the potential influence of confounders (age, sex, disease duration, education, LEDD) on the cognitive change score.

As outcome we used cognitive change: this was calculated for each participant as the difference between their baseline and 3 years composite cognitive z scores. This approach was already described elsewhere (Charvet et al., 2014).

We analyzed risk factors in a univariate as well as in a multivariable way.

In order to check the added value of the axial motor signs a series of hierarchical multiple regression models were performed. After determining the unique variance of global cognitive change attributable to global theta power was determined, the score of axial impairment and limb rigidity, respectively, the UPDRS-III were entered into the model.

We compared the following regression models: In model 0 the resulting confounding factors obtained from stepwise regression modeling as well as the global median theta power were included as potential predictors on the cognitive change score. In model 1 UPDRS-III was entered into the model; in model 2 UPDRS-III was replaced by the score of axial impairment and limb rigidity.

For the multivariate models we calculated Akaike Information Criterion, Mean Absolute Percentage Error, and the variance explained (the last one with Lindemann, Merenda and Gold metrics), multiple *R*^2^ (coefficient of determination), change of multiple *R*^2^ and F-statistics. For statistical calculations we used free software environment R, release 3.5.3, with RStudio interface (R Core Team, 2017).

### Cohort

The sample comprised 47 subjects, males 60%. Baseline characteristics of the sample are presented in Table 2.

**Table 2:** Baseline characteristics of the patients.

**Characteristics**	**Median [min, max]**
Age, years	66 [47, 80]
Education, years	15 [9, 20]
Disease duration, years	6 [1, 24]
LEDD, mg/day	507.5 [0, 2,130]
UPDRS-III	14 [2, 36]
Score of axial impairment and limb rigidity	5 [0, 18]
Global theta band power (median)	0.21 [0.09, 0.53]

*LEDD, Levodopa equivalents of daily dose*.

## Results

According to the Kolmogorov–Smirnov test, all variables analyzed in the regression models were normally distributed.

### Cognitive Change

**Figure 1:**
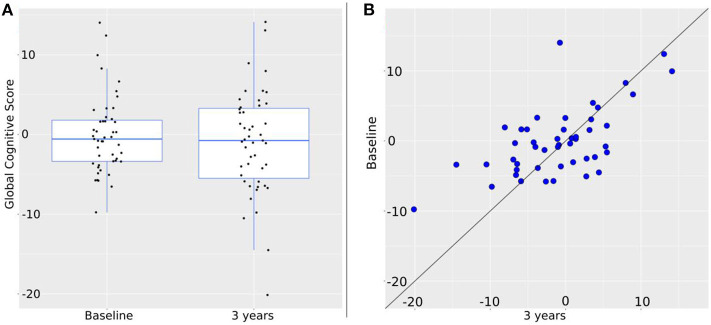
Change of the global cognitive score over 3 years. **(A)** Intra-group change. Global cognition at baseline is: −0.57 [−9.76, 14.03]; at 3 years: −0.76 [−20.14, 14.12]. Black dots represent individual values. **(B)** Individual changes. Blue dots represent global cognition at baseline (vertical axis) and at 3 years (horizontal axis). Therefore, the closer to the diagonal line a dot is, the less change between baseline and 3 years took place. By shift to the vertical axis values at 3 years are worse than at baseline; by shift to the horizontal axis values at 3 years are better than at baseline.

Composite cognitive z scores of the sample at baseline and follow-up, and cognitive change are presented in Figure 1.

### Correlation Matrix Between Variables of Interest

Table 3 shows the intercorrelations of the predictors. Most importantly, no significant correlation between global theta power and axial motor signs was found, indicating independency of the two factors.

**Table 3:** Correlation Matrix.

	**Global theta power**	**Axial motor signs**	**Age**	**Education**	**Disease duration**	**LEDD**	**UPDRS-III**
Global theta power	1						
Axial motor signs	0.10	1					
Age	0.35^**^	0.27	1				
Education	0.39^**^	0.30^*^	0.13	1			
Disease duration	0.28^*^	0.12	0.14	0.11	1		
LEDD	−0.01	−0.18	−0.24	−0.05	0.54^**^	1	
UPDRS-III	0.03	0.90^**^	0.30^**^	0.21	0.21	−0.10	1

Baseline Variables are shown. Significant correlations are labeled with bold and asterisk

* p < 0.05;

** *p < 0.001; LEDD, Levodopa equivalents of daily dose; UPDRS-III, Unified Parkinson's disease rating scale, subscale III*.

### Prediction of Cognitive Change - Regression Models

By stepwise elimination, the following confounders dropped from the model: age, sex, disease duration, and LEDD. Only the parameter education remained in the model explaining cognitive change. Global theta power, score of axial impairment and limb rigidity, and education significantly predicted cognitive change in univariate models (Table 4).

**Table 4:** Univariate models.

**Predictors**	**Beta-estimate; *p*-value of predictors**	**Adjusted R2; *p*-value of the model**	**Akaike's Information Criterion**	**Mean Absolute Percentage Error**
Education, years	−0.52; 0.03	0.07; 0.03	288.62	59%
Axial motor signs	−0.38; 0.03	0.09; 0.03	287.3	40%
Global theta power	−3.20; 0.02	0.11; 0.03	286.49	24%

### Prediction of Cognitive Change – UPDRS-III vs. Axial Motor Signs

The model with the variable axial motor signs had better characteristics than model with UPDRS-III (Table 4). Difference of cognitive change between baseline and follow-up used as dependent variable.

The conducted hierarchical regression model showed that axial motor signs is a significant predictor and increased the prediction of cognitive change with a trend toward significance (*p* = 0.06). Overall, severer cognitive decline at 3 years follow up is significantly predicted by higher theta power values and more impaired axial motor signs at baseline (Table 5).

**Table 5:** Multivariate models.

**Models**	**Adjusted R2 of the model**	**R2-change, *p*-value**	***p*-value of the model**	**Akaike Information Criterion**	**Mean Absolute Percentage Error**	**Variance explained of the model**	**Beta-estimate; *p*-value; explained variance of the predictors**
**Model 0**, predictors: Global theta power, Education	0.12	-	0.01	288.67	24%	16%	**Global theta power:** −2.56; *0.04; 10.24%
							**Education**: −0.32; 0.19; 6.2%
**Model 1**, predictors: Global theta power, Education, UPDRS-III	0.16	0.047; 0.11	0.02	285.96	49%	21.16%	**Global theta power:** −2.62;* 0.03; 10.23%
							**UPDRS-III:** −0.09; 0.06; 5,81%
							**Education:** −0.24; 0.05; 5.10%
**Model 2**, predictors: Global theta power, Education, axial motor signs	0.18	0.067; 0.06	0.009	284.76	31%	23.14%	**Global theta power:** −2.58; *0.03; 9,91%
							**Axial motor signs:** −0.30; 0.03; 8.71%
							**Education:** −0.18; 0.09; 4.50%

*Model 0 was compared to Model 1 and to Model 2. UPDRS-III, Unified Parkinson's disease Rating Scale, subscale III*.

## Discussion

Firstly, we replicated the previous findings, that the severity of axial motor impairment in PD can predict cognitive decline. Secondly, the axial motor signs and global theta power do not correlate with each other, thus, suggesting to provide statistically non-redundant information the risk of cognitive decline in PD.

One theory explaining the association of axial motor dysfunction and cognitive decline is that PD patients with a greater risk of cognitive decline exhibit greater cholinergic deficits at pedunculo-pontine nucleus and nucleus basalis of Meynert, whereas cholinergic deficits are also at the basis of cognitive decline (Levy et al., 2000; Gratwicke and Foltynie, 2018). This theory is supported by studies showing that the risk of severe cognitive decline is associated with akinetic-rigid subtype of PD (Williams-Gray et al., 2007), speech and gait impairment, and postural instability (Alves et al., 2006; Burn et al., 2006), and progression of axial impairment in UPDRS-III (Gago et al., 2009).

Interestingly, akinetic-rigid type of motor impairment is higher correlated with cognitive decline in PD than is general motor impairment (Gago et al., 2009; Vasconcellos and Pereira, 2015).

EEG slowing assessed with quantitative methods predicts cognitive decline in PD, various research groups showed it (Klassen et al., 2011; Caviness et al., 2015).

One particularly interesting finding of this study is that higher education (more years of education) predicts cognitive worsening in PD (Table 3). Indeed, the role of education as a risk factor for cognitive impairment is still debated: lower education was observed to be a risk factor of cognitive impairment (Reuser et al., 2011; Contador et al., 2017), but, at the same time, high education has been found to lead to more rapid cognitive decline (Meng and D'Arcy, 2012; Contador et al., 2017). However, in our study, this effect was not stable in multivariate regression analysis, and, perhaps, needs further focused investigation.

We realize that clinics may be using different kinds of EEG recording systems, and the differences in the number of electrodes, as well as in the recording and processing protocols may influence the time taken, along with variations in the nuanced results. Using a high-density electrode system allows us to pick up signals from a larger area of the brain and aggregate signals from nearby locations, which can often lead to significant noise reduction.

These systems have specially been found useful in detecting spikes in epileptic patients, and while applying source localization techniques (Michel and Brunet, 2019).

In addition, there are several other limitations in this study, which should be addressed in future research. First, the relatively small sample size and potential recruitment bias (via outpatient clinics rather than a community based sample) may limit the generalizability of the results. Second, given the correlational nature of these findings, larger cohorts with longer observation and various assessment tools are warranted to make firmer causal and developmental inferences. Despite these and other limitations, this study has strengths, which include its prospective design, assessing cognitive changes with a cognitive test battery, with all test variables scaled to a normative database, and combining neurophysiological (qEEG) and clinical (UPDRS-III) methods. The findings of this study (a) provide partial support for akinetic-rigid subtype of PD as a risk factor of cognitive decline, and (b) contribute to the search of potential candidates to composite marker of cognitive decline in PD.

The assessment of axial impairment and limb rigidity in combination with qEEG may improve early identification of PD patients prone to severe cognitive decline.

## Data Availability Statement

The datasets generated for this study are available on request to the corresponding author.

## Ethics Statement

The studies involving human participants were reviewed and approved by Ethikkommission beider Basel ref. no.: 135/11. The patients/participants provided their written informed consent to participate in this study.

## Author Contributions

VK: execution of the research project, statistical analysis, writing the first draft, manuscript review and critique. MC: statistical analysis. UG: organization of the research project, statistical review and critique, manuscript review and critique. FH: execution of the research project. AM: execution of the research project, manuscript review and critique. VR: statistical review and critique. PF: conception, organization and execution of the research project, statistical review and critique, and manuscript review and critique. All authors contributed to the article and approved the submitted version.

## Conflict of Interest

The authors declare that the research was conducted in the absence of any commercial or financial relationships that could be construed as a potential conflict of interest.
